# Mufangji Decoction and Its Active Ingredient Patchouli Alcohol Inhibit Tumor Growth through Regulating Akt/mTOR-Mediated Autophagy in Nonsmall-Cell Lung Cancer

**DOI:** 10.1155/2021/2373865

**Published:** 2021-11-02

**Authors:** Liu Yang, Hongyu Chen, Ruixiao Li, Haoze Li, Xing Rui, Lihong Zhou, Ningning Liu, Qing Ji, Qi Li

**Affiliations:** ^1^Department of Oncology, Baoshan Branch, Shuguang Hospital Affiliated to Shanghai University of Traditional Chinese Medicine, Shanghai, China; ^2^Department of Medical Oncology and Cancer Institute of Integrative Medicine, Shuguang Hospital, Shanghai University of Traditional Chinese Medicine, Shanghai, China; ^3^Academy of Integrative Medicine, Shanghai University of Traditional Chinese Medicine, Shanghai, China

## Abstract

**Background:**

Nonsmall-cell lung cancer (NSCLC) is the main type of lung cancer, whose morbidity and mortality rank first among malignant tumors. More than 70% of NSCLC patients are diagnosed at locally advanced or advanced stage, missing the best operation period. Chemotherapy and targeted therapy are important means for the treatment of advanced NSCLC, but various side effects seriously affect the curative effect and the life quality of NSCLC patients. Our previous clinical practice has shown that Mufangji Decoction, a classic traditional Chinese medicine, has a significant curative effect in the treatment of NSCLC, but the specific mechanism is not clear. This study intends to explore the potential mechanism of Mufangji Decoction and its active ingredient patchouli alcohol against NSCLC and to provide a scientific basis for the prevention and treatment of NSCLC by traditional Chinese medicine.

**Methods:**

The in vivo and in vitro experiments were performed to evaluate the antitumor effects and investigate the underlying mechanism of Mufangji Decoction and its active ingredient patchouli alcohol. Network pharmacology was applied to analyze the effective ingredients and potential targets or signaling pathways of Mufangji Decoction.

**Results:**

Our current study shows that Mufangji Decoction can effectively inhibit the growth of subcutaneous transplantation of NSCLC. The following network pharmacological analysis and in vivo experiment suggest that patchouli alcohol is one of the main active ingredients of Mufangji Decoction and exerts antitumor effects. Further mechanism investigation reveals that the antitumor effect of patchouli alcohol is related to the induction of Akt/mTOR signaling pathway-mediated autophagy in NSCLC cells.

**Conclusion:**

Mufangji Decoction and its active ingredient patchouli alcohol might exert their antitumor effects in NSCLC partly through regulating Akt/mTOR-mediated autophagy, providing the evidence that traditional Chinese medicine might be a key approach for NSCLC treatment via targeting the Akt/mTOR signal axis.

## 1. Background

Lung cancer is a malignant tumor that seriously endangers human health, and its morbidity and mortality rank first in malignant tumors [1, 2]. Lung cancer is divided into nonsmall-cell lung cancer (NSCLC) and small cell lung cancer (SCLC) according to its pathological characteristics. Among them, NSCLC accounts for about 80%–90%. Lung cancer of early stage adopts surgery-based treatment, but more than 70% of patients are locally advanced or advanced when diagnosed, leading to extremely poor prognosis [3, 4]. Traditional chemotherapy has poor selectivity for tumor cells, with an effective rate of only 20%–30%. While destroying tumor cells, chemotherapy also causes certain damage to the normal cells, bringing about various adverse reactions and seriously affecting the quality of life of patients [5, 6]. Therefore, finding more effective treatment methods has become the primary problem that needs to be solved urgently in the treatment of lung cancer.

Long-term clinical practice shows that traditional Chinese medicine is playing an increasingly important role in the treatment of lung cancer and other malignant tumors. A classic Chinese medicine compound, Mufangji Decoction, is composed of Mufangji (Radix Cocculi Trilobi), Guizhi (Cassia Twig), Renshen (Ginseng), and Shigao (Gypsum). In recent years, studies have shown that Mufangji Decoction has a good effect in treating phlegm, cough, asthma, and cancerous pleural fluid [7]. Our previous clinical practice also suggested that Mufangji Decoction has a significant curative effect in the treatment of NSCLC, but the underlying mechanism is not clear. Therefore, this study intends to use in vivo and in vitro experiments, combined with network pharmacology, to explore the potential mechanism of Mufangji Decoction and its active ingredients against NSCLC and to provide a scientific basis for the prevention and treatment of NSCLC by traditional Chinese medicine.

## 2. Methods

### 2.1. Cell Culture

Human lung cancer A549 cells were purchased from ATCC (USA) and cultured in the RPMI1640 medium containing 10% fetal bovine serum in a humidified, 5% CO_2_, 37°C incubator.

### 2.2. Preparation of Drugs

Mufangji Decoction is composed of Mufangji (Radix Cocculi Trilobi), Guizhi (Cassia Twig), Renshen (Ginseng), and Shigao (Gypsum). The quality control result of Mufangji Decoction (MFJD) was provided in Supplementary Materials (available here). According to the composition ratio of the compound, the concentration of crude drug is adjusted after decoction and precipitation. The concentration of Mufangji Decoction was divided into three concentrations—0.3 g/mL, 0.6 g/mL, and 1.2 g/mL—and then stored in a refrigerator at 4°C for later use. For in vivo experiments, patchouli alcohol was prepared as follows: patchouli alcohol standard crystals are dissolved in saline containing 5% DMSO, and the concentration of patchouli alcohol was set as 5 mg/kg (low dose), 10 mg/kg (medium dose), and 15 mg/kg (high dose). For in vitro experiments, patchouli alcohol was prepared as follows: patchouli alcohol standard crystals are dissolved and diluted with DMSO into a mother concentration of 200 mmol/L, and then it was stored in a refrigerator at 4°C. The final concentration of DMSO should be less than 0.1% during administration. The concentration settings of patchouli alcohol for cell experiments: 50 *μ*g/mL (low dose), 75 *μ*g/mL (medium dose), and 100 *μ*g/mL (high dose).

### 2.3. Western Blot

A549 cells with different treatments were lysed and then were centrifuged at 4°C and 12,000 r/min. The collected supernatant was quantified. 40 *μ*g protein sample was added to the protein loading buffer and denatured. Then, each pretreated sample was subjected to polyacrylamide gel protein electrophoresis and was transferred to the PVDF membrane. The protein transfer membrane was blocked with a 5% BSA blocking solution for 2 h at room temperature followed by primary antibody and secondary antibody treatment. The main antibodies used are p-Akt (CST, USA), Akt (CST Reagents, USA), p-mTOR (CST, USA), mTOR (CST, USA), LC3-II/I (CST, USA), beclin-1 (CST, USA), p-AMPK*α* (CST, USA), p-p38-MAPK (CST, USA), PCNA (CST, USA), and GAPDH (CST, USA). The secondary antibody was HRP-labeled goat anti-rabbit/mouse IgG. Quantitative analysis was performed using Quantity One software, and the GAPDH expression in the same lane was normalized. All determinations are performed in triplicate and independently repeated at least three times.

### 2.4. CCK-8 Assay

A549 cells in the logarithmic growth phase were seeded in a 96-well plate with 5 × 10^3^ cells per well. After overnight culture, cells were refreshed with the medicated medium, and the concentrations of patchouli alcohol were 0, 25, 50, 75, 100, and 200 *μ*g/mL. For each group, 6 duplicate wells were prepared. After 24 h, 10% (v/v) of CCK-8 solution was added to each well and mixed thoroughly. After incubation at 37°C for 4 h, the absorbance (A) at 490/630 nm (dual wavelength) was detected using a microplate reader. Cell survival rate (%) = [(A experimental group − A blank group)/(A negative control group − A blank group)] × 100%.

### 2.5. Construction of A549-mRFP-GFP-LC3 Cell Line

A549 cells in the logarithmic growth phase were seeded in 96-well plates with 5 × 10^3^ cells per well. The mRFP-GFP-LC3 lentivirus infected A549 cells with MOI of 100 : 1. Simultaneously, 10 pg/mL polybrene was added to facilitate lentivirus transfection efficiency. After 72 h, the transfection efficiency was determined via a fluorescence microscope to ensure that over 95% of green fluorescent-labeled cells were visible. Then, a single-cell clone was picked up and transferred to a 24-well plate for cell proliferation. After 3 or more times of passages, the proportion of fluorescently labeled cells was stable. When the fluorescently labeled cells accounted for 95%, the cell line A549-mRFP-GFP-LC3 stably expressing LC3 fluorescent protein was obtained.

### 2.6. Autophagy Detection

The A549-mRFP-GFP-LC3 cell lines in the logarithmic growth phase were seeded into 24-well plates with 5 × 10^4^ cells per well. Mediums containing patchouli alcohol at the final concentration of 100 *μ*g/mL and 0.5% DMSO were added. After 24 h, fluorescence aggregation was observed under a fluorescence microscope (400×). The degree of autophagy was determined according to the phenomenon of LC3 fluorescence aggregation (yellow spot) in which GFP (green fluorescence) and RFP (red fluorescence) overlap. 5 fields of vision were randomly selected to calculate the proportion of autophagy in total cells.

### 2.7. Animal Experiment

BALB/c nude mice (4–6 weeks old, 18–20 g) were purchased from Shanghai Slack Experimental Animal Co., Ltd., and reared in separate cages in the Experimental Animal Center of the Shanghai University of Traditional Chinese Medicine. A549 cells were inoculated at a cell density of 2 × 10^6^ cells/100 *μ*L to establish a nude mouse subcutaneous tumor model. After 10 days of inoculation, the mice were treated with drugs. After 42 days, the mice were euthanized, and the tumors were stripped and fixed with paraformaldehyde for subsequent testing. All animal experiments were carried out in accordance with the National Animal Research Guidelines and were approved by the Ethics Committee of Shuguang Hospital, Shanghai University of Traditional Chinese Medicine.

### 2.8. Immunohistochemistry

The paraformaldehyde-fixed tumor body is sequentially processed through the steps of dehydration, embedding, antigen retrieval, inactivation of endogenous enzyme activity, antibody incubation, color development with dye, and nucleus staining. The finished film is photographed under a microscope.

### 2.9. Statistical Analysis

The statistical data are expressed as mean ± SD, using SPSS 25.0 software, and one-way analysis of variance was used to compare the differences between each group. *P* < 0.05 indicates that the difference is statistically significant.

## 3. Results

### 3.1. Mufangji Decoction Inhibits the Growth of Subcutaneous Transplantation of NSCLC

**Figure 1:**
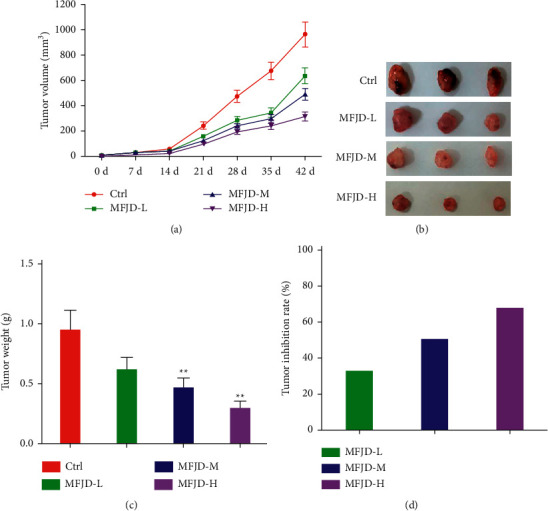
Effect of Mufangji Decoction on the growth of subcutaneous transplantation of NSCLC. (a) The effect of different doses of Mufangji Decoction on the volume of subcutaneously transplanted NSCLC. (b, c) After the intervention of different doses of Mufangji Decoction, the subcutaneously transplanted tumor was surgically removed and the tumor size in each group was weighed. (d) Comparison of the tumor inhibition rate of different doses of Mufangji Decoction. MFJD-L: 0.3 g/mL, MFJD-M: 0.6 g/mL, MFJD-H: 1.2 g/mL. Compared with the model group,  ^*∗∗*^*P* < 0.01.

First, we investigated the inhibitory effect of the traditional Chinese medicine Mufangji Decoction on the growth of subcutaneous transplantation of NSCLC. We set three doses of Mufangji Decoction—low, medium, and high—to verify the antitumor effect of Mufangji Decoction in vivo. Experimental results show that, after different doses of Mufangji Decoction were administered to the stomach for 42 days, the three doses of Mufangji Decoction can inhibit the growth of subcutaneously transplanted tumors in a dose-dependent manner (Figure 1(a)). After intragastric administration for 42 days, we surgically removed the subcutaneously transplanted tumors in each group and weighed the tumor body weight. The results showed that the three doses of Mufangji Decoction reduced the weight of NSCLC subcutaneously transplanted tumors in a dose-dependent manner (Figures 1(b) and 1(c)). In which, the tumor inhibition rate was 33.64% in the low-dose group, 55.27% in the medium-dose group, and 62.47% in the high-dose group (Figure 1(d)). In addition, in the experiment, we have also checked the side effect of Mufangji Decoction in the NSCLC mice model, including losing weight, hepatotoxicity, renal failure, etc., and no side effect was observed. The above research results show that Mufangji Decoction can inhibit the growth of NSCLC subcutaneously transplanted tumors in a dose-dependent manner.

### 3.2. Network Pharmacology Analysis of Mufangji Decoction

**Figure 2:**
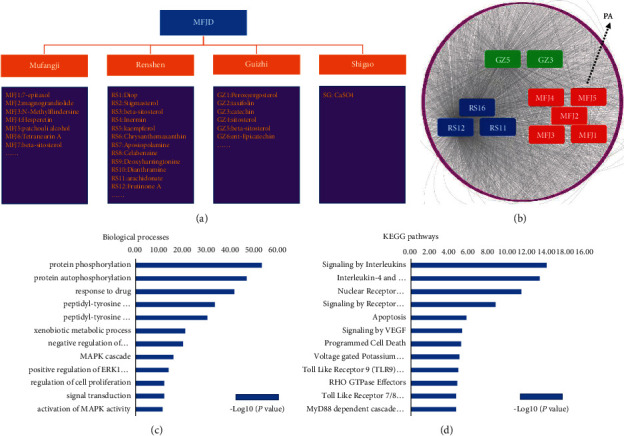
Network pharmacology analysis of Mufangji Decoction. (a) Screening diagram of key active ingredients of Mufangji Decoction. (b) Using the DrugBank database to identify the main targets of the key active ingredients of Mufangji Decoction. (c, d) GO function and KEGG pathway enrichment analysis of the biological processes involved in the key targets of the active ingredients of Mufangji Decoction.

According to network pharmacology-related databases (including Cancer HSP, TCMSP, TCMID, TCM-PTD, TCM Database@Taiwan), combined with the principles of OB>30%, DL > 0.18, etc., the active ingredients of Mufangji Decoction were screened. The results showed that there were 26, 219, and 190 compounds in Mufangji, Guizhi, and Renshen, respectively, and was only 1 compound in Shigao. The main active ingredients include 7-epitaxol, magnograndiolide, N-methylflindersine, hesperetin, beta-sitosterol, patchouli alcohol, tetraneurin A, etc. (Figure 2(a)). For these active ingredients, we used the DrugBank database to identify their targets and screened out a total of 2772 targets (Figure 2(b)). For these targets, GO functional analysis suggests that the key targets of Mufangji Decoction are mainly related to protein phosphorylation, drug response, regulation of cell proliferation, and regulation of MAPK and ERK signals (Figure 2(c)). KEGG pathway analysis found that the key targets of Mufangji Decoction are mainly related to biological regulation processes such as interleukin-mediated signal pathways, apoptosis regulation, programmed cell death regulation, and nuclear receptor transcription pathways (Figure 2(d)).

### 3.3. Patchouli Alcohol Inhibits the Growth of NSCLC Cells

**Figure 3:**
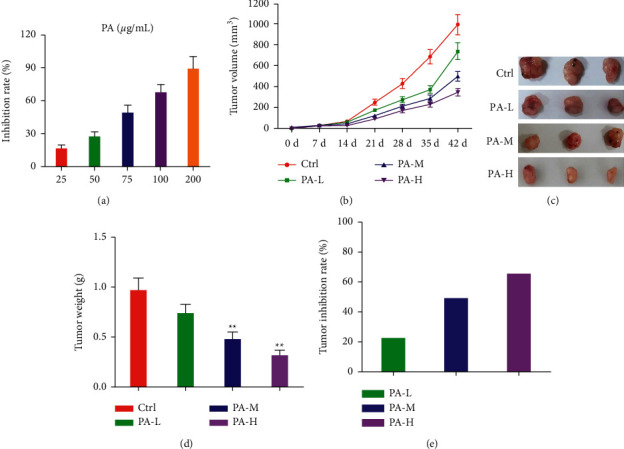
In vivo and in vitro experiments of patchouli alcohol, an effective ingredient of Mufangji Decoction, on the growth of NSCLC. (a) The effect of different doses of patchouli alcohol on the proliferation of NSCLC cells A549. (b) The effect of different doses of patchouli alcohol on the volume of subcutaneously transplanted NSCLC. (c, d) After the intervention of different doses of patchouli alcohol, the subcutaneously transplanted tumor was surgically removed and the tumor size of each group was weighed. (e) Comparison of the tumor inhibition rate of different doses of patchouli alcohol. PA-L: 5 mg/kg; PA-M: 10 mg/kg; PA-H: 15 mg/kg. Compared with the model group,  ^*∗∗*^*P* < 0.01.

According to above network pharmacological analysis results, as well as our quality control results of Mufangji Decoction in Supplementary Materials, patchouli alcohol is an important active ingredient of Mufangji Decoction. Combining relevant literature [8], we next focus on studying whether patchouli alcohol has an inhibitory effect on the proliferation of NSCLC cells and the growth of NSCLC subcutaneous xenografts. In the in vitro experiment, the concentration of patchouli alcohol was set as 25 *μ*g/mL, 50 *μ*g/mL, 75 *μ*g/mL, 100 *μ*g/mL, and 200 *μ*g/mL to treat lung cancer A549 cells for 48 hours. CCK-8 results showed that patchouli alcohol had a significant inhibitory effect on the proliferation of A549 cells in a dose-dependent manner (Figure 3(a)). In the in vivo experiment part, patchouli alcohol was divided into low (5 mg/kg), medium (10 mg/kg), and high (15 mg/kg) doses for intraperitoneal injection of NSCLC-transplanted mice. The results of animal experiments showed that compared with the control group, all the low, medium, and high doses of patchouli alcohol can inhibit the growth of subcutaneously transplanted tumor volume in a dose-dependent manner (Figure 3(b)). After 42 days of intragastric administration, the subcutaneously transplanted tumors in each group were surgically removed and the tumor weights were weighed. The results showed that compared with the control group, all the low, medium, and high doses of patchouli alcohol reduced the weight of NSCLC subcutaneously transplanted tumors in a dose-dependent manner (Figures 3(b) and 3(c)). The tumor inhibition rate was 23.48% in the low-dose group, 50.05% in the medium-dose group, and 66.32% in the high-dose group (Figure 3(d)). Additionally, the side effect of patchouli alcohol in the NSCLC mice model was also checked, and no side effect was observed. The above research results show that patchouli alcohol, the active ingredient of Mufangji Decoction, can inhibit the growth of NSCLC subcutaneous transplantation tumors in a dose-dependent manner.

### 3.4. Patchouli Alcohol Induces Autophagy of NSCLC Cells In Vitro

**Figure 4:**
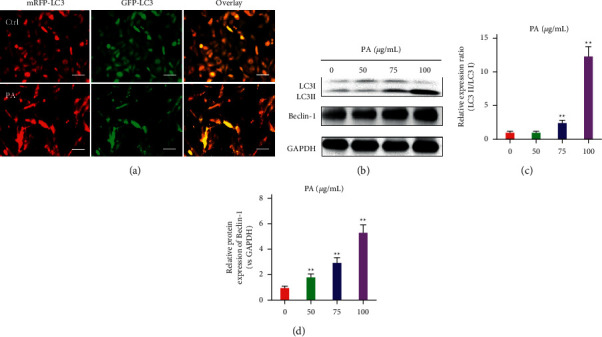
Patchouli alcohol induces autophagy of lung cancer cells A549. (a) After A549 cells were treated with 100 *μ*g/mL patchouli alcohol for 48 hours, the formation of autophagic spots was observed under a fluorescence microscope. (b–d) Western blot and quantitative assay for the key markers of autophagy: LC3-II, LC3-I, and beclin-1. Compared with the control group,  ^*∗∗*^*P* < 0.01.

The mCherry-GFP-LC3 recombinant adenovirus can effectively express the fusion protein of red fluorescent protein mCherry, green fluorescent protein GFP, and LC3B in the target cells after infecting the cells, showing bright red and green fluorescence. In order to verify whether A549 cells undergo autophagy after treatment with patchouli alcohol, the A549 cells were infected with mCherry-GFP-LC3 recombinant adenovirus, and then the A549 cells were intervened with patchouli alcohol for 48 hours to observe whether autophagy spots were formed under a fluorescence microscope. After treating the A549 cells with 100 *μ*g/mL patchouli alcohol for 48 hours, mCherry-GFP-LC3 was found to accumulate on the autophagosome membrane under a fluorescence microscope, which appeared in the form of green spots and red spots, and the fusion became yellow spots (Figure 4(a)). Furthermore, we used western blot to detect the expression of the key markers of autophagy, LC3-II/I and beclin-1. The results showed that patchouli alcohol can upregulate the ratio of LC3 protein II/I and the expression of beclin-1 in the lung cancer cell A549 (Figures 4(b)–4(d)). The above in vitro experiments confirmed that patchouli alcohol can induce autophagy of NSCLC A549 cells.

### 3.5. Patchouli Alcohol Induces Autophagy of NSCLC Cells In Vivo

**Figure 5:**
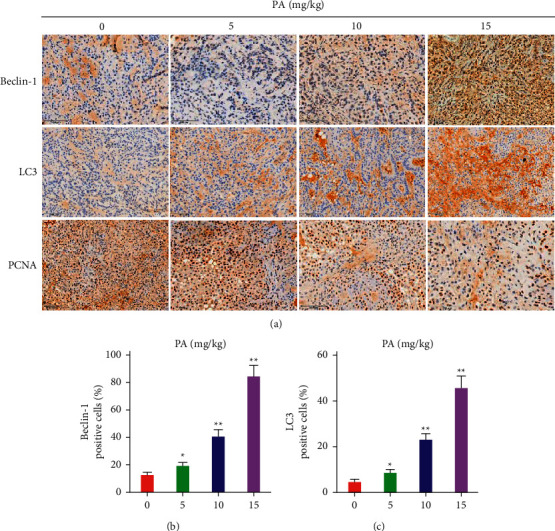
Patchouli alcohol induces autophagy of NSCLC cells in subcutaneously transplanted tumors. (a) The effect of different doses of patchouli alcohol on the expression of beclin-1, LC3, and PCNA protein in subcutaneously transplanted lung cancer tissues. (b, c) Quantitative graph of beclin-1 and LC3 protein positive expressing cells in subcutaneously transplanted NSCLC tissues with treatment of different doses of patchouli alcohol. Compared with the model control group, ^*∗*^*P* < 0.05,  ^*∗∗*^*P* < 0.01.

Early in vitro experiments have shown that patchouli alcohol can induce autophagy of lung cancer A549 cells, but the effect of patchouli alcohol on NSCLC subcutaneously transplanted tumors in vivo is not clear. Therefore, the next step is to detect the expression of autophagy-related proteins LC3, beclin-1, and proliferation-related protein PCNA in subcutaneous xenograft tissues of NSCLC under the treatment of different doses of patchouli alcohol. The experimental results showed that compared with the model control group, the expression of autophagy-related proteins LC3 and beclin-1 were significantly upregulated and that of the proliferation-related protein PCNA was significantly downregulated, which were consistent with the effects of the previous cell experiment (Figures 5(a)–5(c)), further confirming the induction of NSCLC cells autophagy by patchouli alcohol.

### 3.6. Patchouli Alcohol Regulates Autophagy-Associated Akt/mTOR Signaling Pathway

**Figure 6:**
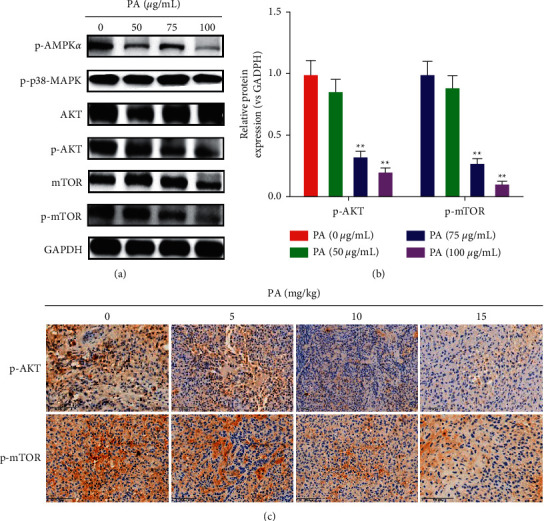
Regulatory effect of patchouli alcohol on the Akt/mTOR signaling pathway in NSCLC cells. (a, b) Western blot and quantitative assay for the protein expression of p-AMPK*α*, p-p38-MAPK, Akt, p-Akt, mTOR, and p-mTOR. GAPDH was used as the control. (c) Immunohistochemistry was performed to detect the effect of patchouli alcohol on the expression of p-Akt and p-mTOR proteins in subcutaneously transplanted tumor tissues of NSCLC. Compared with the control group,  ^*∗∗*^*P* < 0.01.

AMPK, MAPK, Akt/mTOR, and other signaling pathways are currently known to be closely related to autophagy. In order to explore the potential mechanism of patchouli alcohol-induced lung cancer autophagy, we detected the expression of AMPK, MAPK, and Akt/mTOR-related proteins in NSCLC cells treated with patchouli alcohol. The experimental results showed that, compared with the blank control group, the expression of p-Akt and p-mTOR proteins related to the Akt-mTOR signaling pathway was significantly downregulated by patchouli alcohol in a dose-dependent manner (Figures 6(a) and 6(b)), while AMPK and MAPK signaling pathways-associated key proteins p-AMPK*α* and p-p38-MAPK did not change significantly (Figures 6(a) and 6(b)), suggesting that patchouli alcohol-induced NSCLC autophagy may be related to the regulation of Akt/mTOR signaling pathway.

Next, using above NSCLC subcutaneously transplanted tumor tissues, we further verified whether patchouli alcohol has a regulatory effect on AMPK, MAPK, and Akt/mTOR pathways. The results of immunohistochemistry showed that, compared with the blank control group, the expression of p-Akt and p-mTOR protein was significantly downregulated by patchouli alcohol in a dose-dependent manner (Figure 6(c)). However, patchouli alcohol has no obvious regulatory effect on the expression of p-AMPK-*α* and p-p38-MAPK (Figure S1). This result further suggests that patchouli alcohol may induce autophagy of NSCLC cells through the Akt/mTOR pathway.

## 4. Discussion

Presently, the morbidity and mortality of lung cancer in the world and in China rank first among malignant tumors [1, 2, 9]. According to the tumor cell morphology, lung cancer is divided into two categories: NSCLC and SCLC. NSCLC accounts for more than 80% of the incidence of lung cancer in China. Surgery-based treatment is often used in NSCLC of early stage, but more than 70% of NSCLC patients are diagnosed as locally advanced or advanced diseases, leading to the delays in the diagnosis and treatment of NSCLC. Chemotherapy and targeted therapy are important means for the treatment of advanced NSCLC, but various side effects seriously affect the life quality of NSCLC patients.

Long-term clinical practice shows that traditional Chinese medicine (TCM) is playing an increasingly important role in the treatment of lung cancer and other malignant tumors. Several studies have shown that Mufangji Decoction is effective in treating phlegm, cough, and asthma [7], and it is also effective in treating cancerous pleural effusion [10]. Our previous clinical practice has also shown that Mufangji Decoction is effective in the treatment of NSCLC, but the specific therapeutic mechanism is not clear. In this study, we firstly confirmed through in vivo experiments that Mufangji Decoction can inhibit the growth of NSCLC subcutaneously transplanted tumors in a dose-dependent manner. Nevertheless, it is not clear which active ingredients in Mufangji Decoction work and how their mechanism of action works.

TCM and its compound prescriptions have the effects of multiple components and multiple targets. The research in the new field of “traditional Chinese medicine network pharmacology” is based on the theory of systems biology, aims to reveal the mystery of TCM prescriptions at the system level and molecular level, and promote the shift of TCM research mode from the current “single target, single drug” model to a new model of “network target, multicomponent drugs.” Preliminary research results indicate that TCM network pharmacology can provide a new way for TCM to move from experience-based medicine to evidence-based medicine and accelerate the process of TCM drug discovery, while improving current drug research strategies and enabling preliminary analysis of drugs affecting the disease network [11–13]. In this study, we screened the main active ingredients of Mufangji Decoction through network pharmacology and found that patchouli alcohol (PA) is an important active ingredient of Mufangji Decoction. Patchouli alcohol is the main active ingredient of tetrandrine [14], a tricyclic sesquiterpene compound with a molecular formula of C15H26O and a molecular weight of 222.37. Studies have found that patchouli alcohol has significant antitumor effects [15], such as effectively inhibiting the proliferation and inducing the apoptosis of human prostate cancer PC3 cells [16] and inhibiting the proliferation of human lung cancer cell line A549 [4]. In this study, we found through in vivo and in vitro experiments that patchouli alcohol has an obvious inhibitory effect on the growth of NSCLC subcutaneous transplantation tumors and can significantly inhibit the proliferation of NSCLC cells.

Autophagy, also known as type II programmed cell death, refers to the process of cell self-digestion and absorption characterized by autophagosomes wrapped in double-layer membrane vesicles [17]. Autophagy is usually divided into three categories, namely macroautophagy, small autophagy, and chaperone-mediated autophagy [18]. Macroautophagy is also what we commonly call autophagy, which transports substances to be degraded into lysosomes through the formation of autophagosomes [19]. Studies have shown that autophagy-related genes LC3 and beclin-1 are involved in the formation of autophagosomes [20], and the production of LC3 is the most important marker of autophagy [21, 22]. When eukaryotic cells undergo autophagy, the conversion of LC3-I to LC3-II in the cell increases significantly. Our in vitro experiment results showed that patchouli alcohol can induce autophagy of NSCLC cells by upregulating the ratio of LC3 protein II/I and the expression of beclin-1. Moreover, our in vivo experiment further confirmed the effect of patchouli alcohol in inducing NSCLC cells autophagy by upregulating the expression of autophagy-related proteins LC3 and beclin-1 and downregulating the expression of the proliferation-related protein PCNA.

Studies have also shown that multiple signal transduction pathways are involved in the regulation of tumor cell autophagy, such as AMPK, MAPK, Akt/mTOR, and other signal pathways [23–25]. In this study, whether in in vitro or in vivo experiments, patchouli alcohol can induce autophagy in NSCLC cells and has a significant regulatory effect on the key autophagy-related signaling pathway Akt/mTOR, while it has little effect on the phagocytic pathways of AMPK and MAPK, suggesting that patchouli alcohol may induce autophagy in NSCLC cells through the Akt/mTOR signaling pathway. However, in future, more NSCLC cell lines are needed to further validate our data, providing more powerful evidence for the development and application of Mufangji Decoction and patchouli alcohol in NSCLC therapy.

## 5. Conclusion

**Figure 7:**
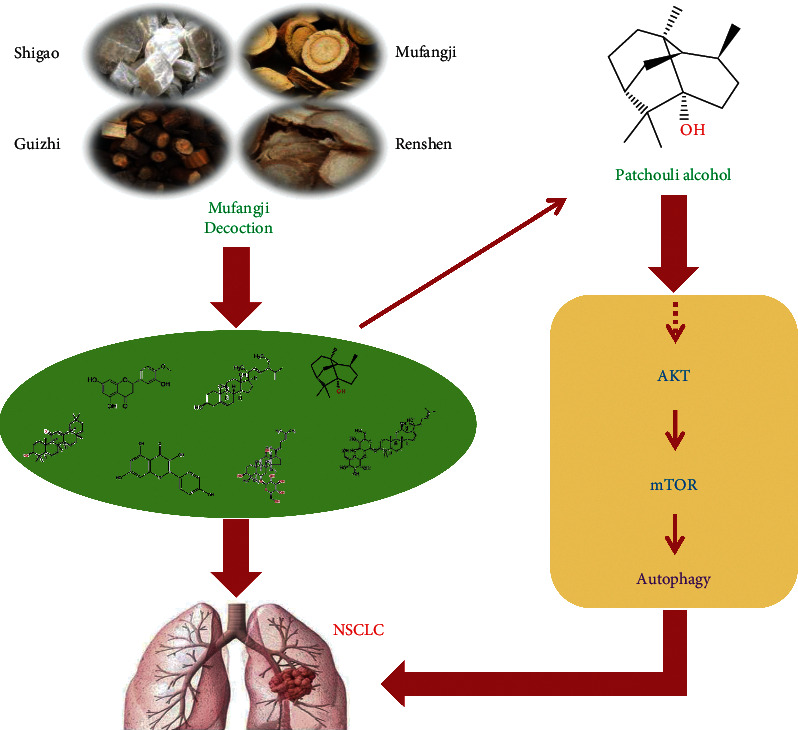
Potential mechanism of Mufangji Decoction and its effective component patchouli alcohol in inducing NSCLC autophagy through the Akt/mTOR signaling pathway.

This study revealed that Mufangji Decoction has a very effective inhibitory effect on the growth of NSCLC subcutaneous transplantation tumors. Furthermore, through network pharmacology, we have screened out one of the important active ingredients of Mufangji Decoction, patchouli alcohol. Further in vivo and in vitro experiments found that the anticancer effect of patchouli alcohol is related to the induction of NSCLC cell autophagy, and its mechanism is related to the regulation of Akt/mTOR signaling pathway (Figure 7). Therefore, targeting the Akt/mTOR signal axis might provide a key approach for the treatment of NSCLC. At the same time, this study provides a preliminary experimental basis for the application of Mufangji Decoction and patchouli alcohol in the clinical treatment of NSCLC.

## Data Availability

The data used to support the findings of this study are included within the article.

## Abbreviations

AKT: Protein kinase B
AMPK: Adenosine 5′-monophosphate (AMP)-activated protein kinase
ATCC: American type culture collection
CCK-8: Cell counting kit-8
DMSO: Dimethyl sulfoxide
ERK: Extracellular regulated protein kinase
GO: Gene ontology
KEGG: Kyoto encyclopedia of genes and genomes
MAPK: Mitogen-activated protein kinase
MFJD: Mufangji decoction
mTOR: Mammalian target of rapamycin
NSCLC: Nonsmall-cell lung cancer
PA: Patchouli alcohol
PVDF: Polyvinylidene fluoride
SCLC: Small cell lung cancer
TCM: Traditional Chinese medicine.

## Supplementary Materials

Supplementary Materials Figure S1. Immunohistochemistry was performed to detect the effect of patchouli alcohol on the expression of p-AMPK*α* and p-p38-MAPK protein in subcutaneously transplanted tumor tissues of NSCLC. Compared with the control group, ^*∗∗*^*P* < 0.01. Determination of quality control components in the compound prescriptions: Mufangji Decoction (MFJD).
