# Supplementary material for: Mufangji Decoction and Its Active Ingredient Patchouli Alcohol Inhibit Tumor Growth through Regulating Akt/mTOR-Mediated Autophagy in Nonsmall-Cell Lung Cancer
Source: Evid Based Complement Alternat Med. 2021 Nov 2;2021:2373865. doi: 10.1155/2021/2373865 (PMC8577897; doi:10.1155/2021/2373865)
Supplement: Supplementary Materials — Figure S1. Immunohistochemistry was performed to detect the effect of patchouli alcohol on the expression of p-AMPKα and p-p38-MAPK protein in subcutaneously transplanted tumor tissues of NSCLC. Compared with the control group, ∗∗P < 0.01. Determination of quality control components in the compound prescriptions: Mufangji Decoction (MFJD). [file 2373865.f1.zip › 2373865.f1/Supplementary materials (4).docx]

**Supplementary materials**

**Supplementary Figure legends**

Figure S1: Immunohistochemistry was performed to detect the effect of patchouli alcohol on the expression of p-AMPKα and p-p38-MAPK protein in subcutaneous transplanted tumor tissues of NSCLC. Compared with the control group, ***P*<0.01.

**Supplementary quality control data**

**Determination of quality control components in the compound prescriptions---Mufangji** **Decoction (MFJD)**

1. Assay Results

1.1 Tetrandrine and Ginsenoside Rg1 Assay

Using UPLC-QqQ/MS in the SIM detection mode to determine the content of Tetrandrine and Ginsenoside Rg1 in the quality control components of Mufangji Decoction (MFJD); Using the standard curve method to plot the relative concentration of the peak area of the analyte, and using the least square method for linear regression analysis, the linear equations of Tetrandrine and Ginsenoside Rg1 are shown in Table 2, the standard curve is shown in Figure 1~Figure 2; The test results are shown in Table 1(Prepare 2 test solution in parallel for each batch of samples, and inject 2 injections of each test solution, denoted as SPL-1-1, SPL-1-2, SPL-2-1, SPL-2-2); The ion current diagram of each channel of the sample and the control is shown in Figure3.

Table 1 The content of Tetrandrine and Ginsenoside Rg1 in the sample

| Compound | Sample | SPL-1-1 | SPL-1-2 | SPL-2-1 | SPL-2-2 | Average content | RSD |
| --- | --- | --- | --- | --- | --- | --- | --- |
| Tetrandrine | NO.1 | 0.0430% | 0.0447% | 0.0431% | 0.0431% | 0.0435% | 1.86% |
|  | NO.2 | 0.0471% | 0.0430% | 0.0425% | 0.0433% | 0.0440% | 4.80% |
| Ginsenoside Rg1 | NO.1 | 0.0428% | 0.0428% | 0.0435% | 0.0412% | 0.0426% | 2.34% |
|  | NO.2 | 0.0434% | 0.0448% | 0.0447% | 0.0452% | 0.0445% | 1.79% |

Table 2 Regression equation, correlation coefficient and linear range of reference substance

| Compound | Regression equation | Correlation r | Linear range(μg/mL) |
| --- | --- | --- | --- |
| Tetrandrine | y = 5.37837*10^6^x + 3.06645*10^6^ | 0.9967 | 0.3719~3.7186 |
| Ginsenoside Rg1 | y = 1.33733*10^5^x + 3406.19730 | 0.9998 | 2.7682~27.6824 |

Figure 1 Standard curve of Tetrandrine reference substance

Figure 2 Standard curve of Ginsenoside Rg1 reference substance

Figure 3 Extraction ion chromatogram of sample and control substance (A: Ion flow diagram of Tetrandrine extracted from sample solution, B: Ion flow diagram of Tetrandrine extracted from control solution, C: Ion flow diagram of Rg1 extracted from sample solution, D: Ion flow diagram of Rg1 extraction from control solution)

1.2 Patchouli alcohol Assay

Determination of Patchouli alcohol in Mufangji Decoction by GC-FID. The external standard one-point method was used to calculate the content of the component to be tested in the sample, and the results are shown in Table 3. The chromatograms of the sample and the control are shown in figures 4 and 5.

Table 3 Contents of Patchouli alcohol in samples

| Compound name | SPL-1-1 | SPL-1-2 | SPL-2-1 | SPL-2-2 | Average content | RSD |
| --- | --- | --- | --- | --- | --- | --- |
| 1 | 0.241% | 0.241% | 0.244% | 0.249% | 0.244% | 1.40% |
| 2 | 0.239% | 0.238% | 0.253% | 0.248% | 0.244% | 2.90% |

Fig. 4 Chromatogram of the control

Fig. 5 Chromatogram of the sample

2.Instruments and Equipment

1290 UPLC, Agilent Technologies;

API 4000 Triple quadrupole mass spectrometer, SCIEX;

One in ten thousand balance ME104, METTLER TOLEDO;

One millionth balance XPR2, METTLER TOLEDO;

3.Reagents and materials

Water: pure water,20201211C,Guangzhou Watson's Food & Beverage Co., Ltd;

Acetonitrile: Mass spectrometry pure,I1099129026,Merck Chemicals (Shanghai) Co., Ltd.;

Formic acid: Mass spectrometry pure,L9620186,Anpel Laboratory Technologies(Shanghai)Inc.;

Hexyl hydride: Analytically pure,P1643191,Shanghai Titan Scientific Co.,Ltd.;

Alcohol: Analytically pure,P1856541,Shanghai Titan Scientific Co.,Ltd.;

Tetrandrine: 99.4%,Batch number: 8309,Shanghai Standard Technology Co., Ltd.;

Ginsenoside Rg1: 90.2%,Batch number: 7925,Shanghai Standard Technology Co., Ltd.;

Patchouli alcohol: 98%, Batch number: 3778, Shanghai Standard Technology Co., Ltd.;

Mufangji: Batch number: 200707,Zhejiang province,Shanghai Kangqiao Chinese Medicine Tablet Co., Ltd.;

Ginseng: Batch number: 210224,Jilin province,Shanghai Hongqiao Chinese Herbal Medicine Co., Ltd.;

Cassia Twig: Batch number: 20210227-1,Shandong province,Shanghai Wanshicheng Pharmaceutical Co., Ltd.;

Gypsum: Batch number: 210219,Hubei province,Shanghai Hongqiao Chinese Herbal Medicine Co., Ltd..

4.Chromatographic and mass spectrometric conditions

4.1 Chromatographic conditions

Chromatograph: Agilent 1290 UPLC;

Chromatographic column: Agilent Poroshell EC-C18(2.1×100 mm,1.9 μm),Lot number: SR047;

Mobile Phase: A: Acetonitrile, B: 0.1% Formic acid-water;

Column temperature: 30℃; Injection volume: 1μl; Velocity of flow: 0.3 ml/min;

Table 3 Mobile phase gradient

| Time (minutes) | Mobile phase A (%) | Mobile phase B (%) |
| --- | --- | --- |
| 0 | 10 | 90 |
| 3 | 10 | 90 |
| 15 | 30 | 70 |
| 15.1 | 95 | 5 |
| 17 | 95 | 5 |
| 17.1 | 10 | 90 |
| 21 | 10 | 90 |

4.2Mass spectrum condition

Table 4 Ion source parameters-positive ion model

| Parameters | Parameter value | Parameters | Parameter value |
| --- | --- | --- | --- |
| Curtain Gas (psi) | 25 | Dwell time (msec) | 100 |
| Ion Source Gas 1 (psi) | 30 | Ion Spray Voltage Floating (V) | 5000 |
| Ion Source Gas 2 (psi) | 30 | Ion Source Temperature (°C) | 450 |

Table 5 Compound SIM parameters

| Serial number | Compound | Ionic state | Precursor Ion(*m*/*z*) | DP(V) | EP(V) |
| --- | --- | --- | --- | --- | --- |
| 1 | Tetrandrine | Cation | 623.3 | 80 | 10 |
| 2 | Ginsenoside Rg1 | Cation | 801.5 | 40 | 10 |

4.3 Gas chromatographic conditions

Detector: Flame ionization detector (FID);

Column: DB-624 Capillary column (The column length is 30m, the inner diameter is 0.25mm, and the film thickness is 1.4μm);

Inlet Temperature: 280 °C;

Detector Temperature: 280 °C;

Program temperature rise: the initial temperature is 110°C, keep for 2min, heat up to 160°C at a rate of 5°C/min', and heat up to 250°C at a rate of 30°C/min, and hold for 10min;

Carrier gas: nitrogen;

Gas flow rate: 1ml/min;

Injection volume: 1μl;

Shunt ratio: 5:1.

5. Preparation of the control solution

Preparation of tetrandrine standard curve solution: Weigh 3.741mg of Tetrandrine reference substance, put it in a 10ml volumetric flask, add 95% ethanol to the mark, and record it as mother liquor. Take 1ml of mother liquor and dilute to a 100ml volumetric flask, record it as Tetrandrine-STD-5; Take 0.7ml of mother liquor and dilute to a 100ml volumetric flask, record it as Tetrandrine-STD-4; Take 1ml of mother liquor and dilute to a 250ml volumetric flask, record it as Tetrandrine-STD-3; Take 1ml of Tetrandrine-STD-5 liquor solution to a 5ml volumetric flask and record it as Tetrandrine-STD-2; Take 1 ml of Tetrandrine-STD-5 liquor and dilute to a 10 ml volumetric flask, record it as Tetrandrine-STD-1. Ultra-high-speed centrifugation (12000 rpm) for 5 minutes, take the supernatant, and obtain the standard curve solution of Tetrandrine of various concentrations.

Weigh 3.069mg of Ginsenoside Rg1 reference substance, put it in a 10ml volumetric flask, add 95% ethanol to the volume, and record it as mother liquor. Take 1ml mother liquid to a 10 ml volumetric flask, record it as Rg1-STD-5; take 0.7ml mother liquid to a 10ml volumetric flask, record it as Rg1-STD-4; take 1ml mother liquid to a volume of 25ml Bottle, record it as Rg1-STD-3, take 1ml Rg1-STD-5 solution to a 5 ml volumetric flask, record it as Rg1-STD-2, take 1ml Rg1-STD-5 solution and dilute to a 10 ml volume Bottle, record it as Rg1-STD-1. High speed centrifugation (12000rpm) 5 minutes, take the supernatant, namely the concentration of Ginsenoside RG1 standard curve solution.

Preparation of Patchouli alcohol reference substance solution: Take an appropriate amount of Patchouli alcohol reference substance, accurately weigh it, add n-hexane to make a solution containing 30 μg Patchouli alcohol per 1 ml, ultra-high-speed centrifugation (12000 rpm) for 5 minutes, and take the supernatant.

6.Preparation of sample solution

The first batch of Mufangji Decoction: Weigh 9.21g of Fangji decoction pieces, 11.74g of Ginseng decoction pieces, 6.07g of Cassia Twig decoction pieces, and 14.99g of Gypsum decoction pieces, put them in a 1000ml round-bottomed flask, add 250ml of 95% ethanol, and weigh it.

The second batch of Mufangji Decoction: Weigh 9.30g of Fangji decoction pieces, 11.95g of Ginseng decoction pieces, 6.07g of Cassia Twig decoction pieces, and 15.05g of Gypsum decoction pieces, put them in a 1000ml round-bottomed flask, add 250ml of 95% ethanol, and weigh it.
