# Supplementary figures and images for: Mufangji Decoction and Its Active Ingredient Patchouli Alcohol Inhibit Tumor Growth through Regulating Akt/mTOR-Mediated Autophagy in Nonsmall-Cell Lung Cancer
Source: Evid Based Complement Alternat Med. 2021 Nov 2;2021:2373865. doi: 10.1155/2021/2373865 (PMC8577897; doi:10.1155/2021/2373865)

**Figure S1**

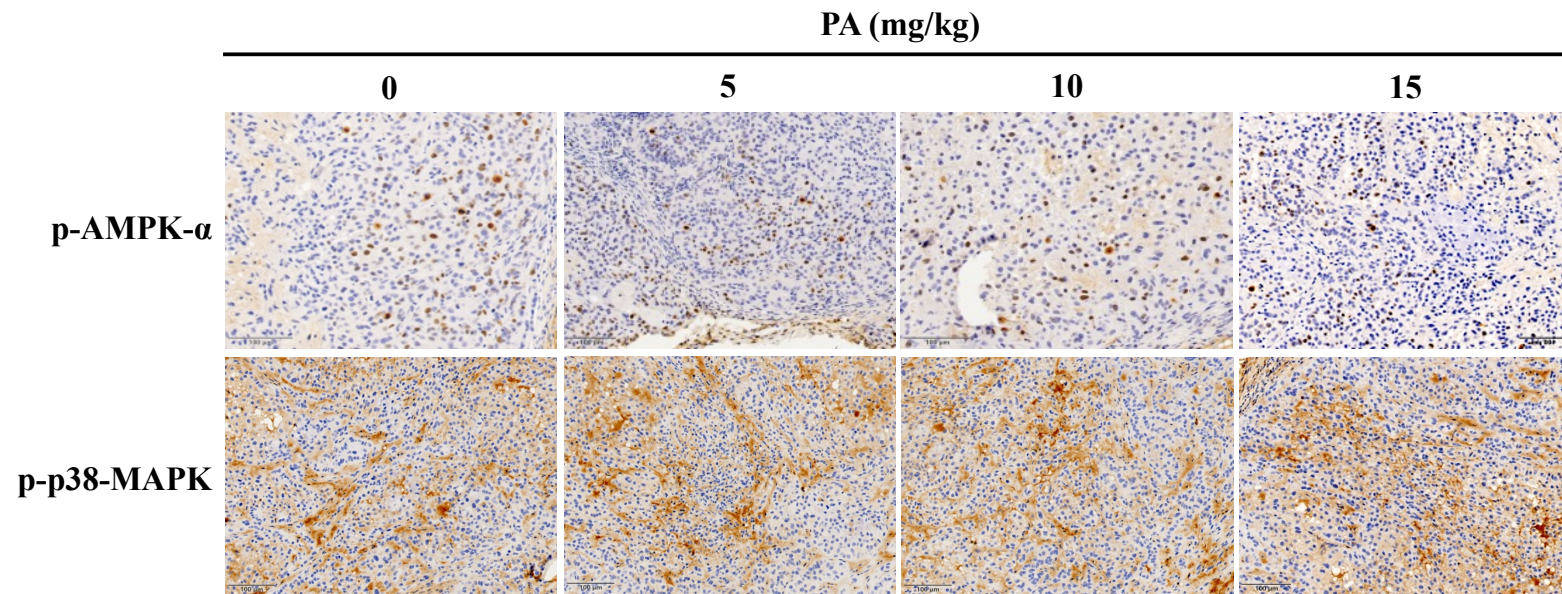

Supplement: Supplementary Materials — Figure S1. Immunohistochemistry was performed to detect the effect of patchouli alcohol on the expression of p-AMPKα and p-p38-MAPK protein in subcutaneously transplanted tumor tissues of NSCLC. Compared with the control group, ∗∗P < 0.01. Determination of quality control components in the compound prescriptions: Mufangji Decoction (MFJD). [file 2373865.f1.zip › 2373865.f1/Figure S1 (1).pdf]
